# Molecular Pathways in Prostate Cancer

**DOI:** 10.5812/numonthly.9430

**Published:** 2013-06-08

**Authors:** Evangelos Mazaris, Alexios Tsiotras

**Affiliations:** 1 Urology Department, Lister Hospital, Stevenage, United Kingdom

**Keywords:** Prostatic Neoplasms, Genes, Therapeutics

## Abstract

**Objectives:**

Prostate cancer is a prevalent disease with a high impact on patients’ morbidity and mortality. Despite efforts to profile prostate cancer, the genetic alterations and biological processes that correlate with disease progression remain partially elusive. The purpose of this study is to review the recent evidence relating to the initiation and progression of prostate cancer in relation to the familial correlation of the disease, the genetic aberrations resulting in prostate cancer and the new molecular biology data regarding prostate cancer.

**Materials and Methods:**

A Medline database search identified all the existing publications on the molecular events associated with the pathogenesis and evolution of prostate cancer. Particular emphasis was given on the specific genetic phenomena associated with prostate cancer.

**Results:**

Like other cancers, prostate cancer is caused by an accumulation of genetic alterations in a cell that drives it to malignant growth. Specific genes and gene alterations have been suggested to play a role in its development and progression. Aneuploidy, loss of heterozygosity, gene mutations, hypermethylation and inactivation of specific tumour suppressor genes such as GSTpi, APC, MDR1, GPX3 and others have been detected in prostate cancers, but generally only at a low or moderate frequency. The androgen receptor (AR) signalling pathway may play a crucial role in the early development of prostate cancer, as well as in the development of androgen-independent disease that fails to respond to hormone deprivation therapies. Other alterations linked to the transition to hormone-independence include amplification of MYC and increased expression of ERBB2 and BCL2. Inflammatory changes may also contribute to the development of prostate cancer.

**Conclusion:**

The identification of specific molecular markers for prostate cancer may lead to its earliest detection and better prediction of its behavior. The better understanding of the molecular events affecting prostate cancer progression may result in the introduction of new drugs to target these events thus providing a potential cure and a tool for prevention of this very common disease.

## 1. Introduction

Prostate cancer is the most commonly diagnosed male malignancy and the second leading cause of cancer death, representing 29% of all male cancer deaths (1). It is usually an indolent disease, but 25-30% of the tumors behave aggressively resulting in almost 30,000 deaths annually in the US (2). In case of metastatic disease, 70-80% of patients, respond initially to androgen-deprivation therapy, but in later stages the tumor becomes hormone-refractory and more aggressive leading to poor prognosis (3).

While localized disease can be effectively treated by various modalities, there is no effective treatment for hormone-refractory disease. The current therapeutic approaches for the advanced stages of prostate cancer are palliative rather than therapeutic.

Thus, determining the molecular pathways that lead to the development and progression of the disease is a challenge. This challenge has been difficult because of the heterogeneity and multifocality of the tumor. The emergence of new investigative tools such as DNA microarray technology and the application of proteomics may enhance our knowledge regarding the initiation, development and progression of prostate cancer. It may become possible to distinguish between indolent and aggressive types reserving radical treatment for the latter ones. Although epidemiologically prostate cancer can be divided in hereditary (4) and sporadic forms, it is not possible to distinguish between these two groups at a molecular level.

## 2. Objectives

The aim of the present study was to review all the existing literature regarding genetic predisposition, somatic alterations, epigenetic changes and in summary, the molecular pathways that lead to prostate cancer.

## 3. Materials and Methods

A thorough Medline search was performed regarding the existing publications on the molecular events related to the pathogenesis and progression of prostate cancer.

## 4. Results

The following genes until today have been implicated in prostate cancer:

A) Hereditary prostate cancer: It is a subtype of familial prostate cancer in which there is a pattern of Mendelian inheritance of a susceptibility gene, however, highly penetrant susceptibility genes causing prostate cancer have not yet been identified (5). On the contrary, multiple genes with a small to moderate effect seem to be involved in prostate cancer carcinogenesis. It seems that the lifetime risk for the development of the tumor increases 2- to 3-fold in men with a first-degree relative with prostate cancer (6, 7). If two or more first-line relatives are affected, the risk increases 5 to 11-fold and when first- and second-degree relatives are affected, prostate cancer risk increases up to 8-fold compared with men with no such family history (7, 8). Furthermore, men with a family history of prostate cancer develop the disease at a younger age (9), usually 6-7 years prior to spontaneous cases (10). Up to 42% of the risk of prostate cancer could be due to heritable factors (11). All three types of inheritance (autosomal dominant, recessive and X-linked) have been reported. Studies have presented an autosomal dominant mode of inheritance accounting for 9% of the total prostate cancer incidence by the age of 85 years (4, 12, 13). Other studies have implied an X-linked or autosomal recessive mode of inheritance for prostate cancer since they observed that men with affected brothers had a higher risk for prostate cancer compared to those with affected fathers (6, 14). Thus, it seems that more than one gene contribute to the inheritance of prostate cancer. Based on family-based linkage analysis, several prostate cancer susceptibility genes have been identified:

a. HPC1 or RNASEL: Located on chromosome 1q24-25 locus it was the first prostate cancer susceptibility gene to be identified (15). RNASEL is a ribonuclease that degrades viral and cellular RNA and can produce apoptosis in viral infection. Until now the RNASEL gene has been identified in many studies as the most important hereditary prostate cancer gene (16-21) although other studies have not supported these findings (22, 23).

b. PCAP: This was the second prostate cancer gene to be identified located on chromosome 1q42.2-43 locus (24, 25).

c. HPCX: This is an X-linked gene located on Xq27-28 (26).

d. CAPB: This gene located on chromosome 1p36 and seems to be important in high-risk prostate cancer families with close relatives suffering from brain tumors (27).

e. HPC20: This is located on chromosome 20q13 and although it was identified as a prostate cancer susceptibility gene (28, 29), others failed to support it (30), however it was suggested that the aforementioned locus may represent a low-penetrance prostate cancer predisposition gene.

f. HPC2/ELAC2: This is located on chromosome 17p. Its function has been proposed as a metal dependent hydrolase. It was suggested as a prostate cancer susceptibility gene (31) but this was not confirmed by subsequent studies (32-34). More recently, it has shown a minor effect, together with RNASEL, in prostate cancer risk in African American familial and sporadic cases (35). The association of one of HPC2/ELAC2 polymorphic variants (Thr541) with prostate cancer seems to be weak (36).

g. Susceptibility locus on 16q23: This was suggested by a large study (37).

h. HSD3B: This prostate cancer susceptibility locus is located on chromosome 1p13 region (38).

i. MSR1: This gene encodes a macrophage scavenger receptor responsible for cellular uptake of molecules, including bacterial cell wall products. Its role in hereditary prostate cancer is controversial since there are those who support it (39, 40) while others have not provided evidence of such a role (41).

j. NBS1: This gene is involved in the rare human genetic disorder, the Nijmegen breakage syndrome, which is characterized by radiosensitivity, immunodeficiency, chromosomal instability and increased risk of lymphatic cancer. It encodes the protein nibrin, which is involved in the processing/repair of DNA double strand breaks and in cell cycle checkpoints (42). Mutations in this gene have been associated with a small increased risk of prostate cancer (43), however one of the founder mutations, i.e 657del5, did not show significant contribution to prostate cancer risk (44).

k. CHEK2: This gene is an upstream regulator of p53 in the DNA damage signaling pathway. Mutations of this gene have been identified in hereditary prostate cancer and they are associated with a small increased risk for the disease (45, 46).

Despite the large number of studies seeking to determine a major prostate cancer susceptibility gene, this has not been achieved yet. Many mutations of the above genes have been identified in sporadic prostate cancer as well. Because of the high frequency of prostate cancer it is probably difficult to distinguish within families cases of sporadic tumors from true hereditary ones. Furthermore, the low penetrance of genes in hereditary prostate cancer may result from the fact that multiple genes are involved in hereditary prostate carcinogenesis exerting a small to moderate effect, which is increased by the proper genetic, dietary and environmental background (47).

B) Sporadic prostate cancer: Most prostate cancers are sporadic including various molecular pathways involved in the initiation, development and spread of the disease:

1) Tumor suppressor genes: The normal gene inhibits the growth of tumor cells. Initially, the loss of function of the gene was attributed to mutation or deletion of the two alleles (48), however, this has been revised to include epigenetic changes such as (a) inactivation of one or both alleles by DNA methylation of CpG sites in gene promoters, (b) heritably downregulated function, (c) function compromised in a clonal fashion (49). Thus, the alteration of normal function can be by mutation, methylation of the promoter or by modification of the protein product (50). Various tumor suppressor genes have been identified, playing a role in prostate cancer development, progression and the emergence of androgen-independent phenotypes:

a. p53 gene: This is the most commonly mutated gene in human cancers. Because of its normal function, which is the prohibition of entrance into the synthetic phase of the cell cycle (phase S) and the promotion of apoptosis in cells that are disorganized or have damaged DNA (5), this gene is recognized as the ‘guardian of the genome’ (1). In primary prostate cancer a relatively low incidence (10-20%) of p53 gene mutations has been described, however, in advanced stages of the disease the p53 is mutated in 42% of the cases (51-53) and it is associated with bone metastases and androgen-independent disease. Abnormal p53 expression correlates with high histological grade, high stage and clinical progression of the disease (5) while, it is also correlated with reduced survival after radical prostatectomy (54) and disease onset modulation (55).

b. PTEN: About 5-27% of localized and 30-60% of metastatic prostate tumors display PTEN mutations (56-58). The PTEN gene encodes a phospholipid phosphatase active against both protein and lipid substrates acting as a tumor suppressor gene by inhibiting the phosphatidyinositol 3-kinase-protein kinase B (PKB-Akt) signaling pathway which is essential for cell cycle progression and cell survival (59). PTEN is present in normal prostatic epithelial cells and in cells with prostatic intraepithelial neoplasia (PIN). In prostatic cancers, PTEN concentrations are reduced particularly in high grade or stage cancers (5). However, it has been shown that common genetic variants in PTEN do not substantially increase the risk of prostate cancer (60). A recent study suggested that germ-line variants in PTEN do not have a significant role in prostate cancer susceptibility (61). PTEN influences the levels of CDKN1B (p27) another tumor suppressor gene.

c. CDKN1B (p27): It is an important tumor suppressor gene in prostate cancer. p27 is a cyclin dependent kinase inhibitor and its reduced levels are common in prostate cancer, especially in more aggressive tumors with a poor prognosis (62-65). This gene is located on chromosome 12p12-3 and the somatic loss of its sequences has been described in 23% of localized prostate cancers, in 30% of regional lymph node metastases and in 47% of distant metastases (66). Low p27 concentrations may be the result of both CDKN1B alterations and as mentioned before because of loss of PTEN function.

d. MX11: This gene is a negative regulator of c-MYC proto-oncogene, which plays a critical role in prostate cancer. There are few reports on the MX11 gene mutations related to prostate cancer (67, 68).

e. NKX3.1: Its product binds to DNA and represses the expression of the PSA gene (69). The loss of function or deletion of this gene appears to be an early event in prostate cancer (5). It is present in androgen-sensitive cells but absent in androgen-independent prostate tumor (70). The loss of this gene may be involved in the increasing concentrations of PSA seen with prostate cancer progression. A study (71) found that NKX3.1 was absent in 20% of PIN lesions, 6% of low stage prostate tumors, 22% of high stage prostate tumors, 34% of androgen-independent tumors and 78% of prostate cancer metastases.

f. Retinoblastoma (Rb) gene: The Rb gene plays an important role in the G1 phase of the cell cycle. Mutations and loss of Rb protein expression have been reported in both clinically localized and advanced prostate carcinomas (72, 73). Studies have demonstrated at least 50% mutations of Rb gene in advanced prostate tumor (74, 75). This gene has also been implicated in regulating apoptosis of prostate cells, especially in response to androgens (76, 77).

g. Glutathione S-transferase gene (GSTP1): It is located in the chromosomal region 11q and it plays a role of a genome caretaker by preventing oxidant and electrophilic DNA damage (77). It has been shown to be inactivated by hypermethylation of the promoter region in prostate cancer (78, 79). Nearly 96% of primary prostatic adenocarcinomas display extensive hypermethylation and thus loss of function of the GSTP1 gene (78), however recent data showed no associated risk for sporadic or familial prostate cancer with polymorphism in codon 105 of the pi variant (GSTP1 I105v) (80). The calculation of GSTP1 hypermethylation can accurately detect the presence of cancer even in small, limited tissue samples. Thus, it is a promising diagnostic marker that could be used as an adjunct to prostate biopsy for prostate cancer screening (81).

h. Kruppel-like factor 6 (KLF6): It is a transcription factor that normally upregulates p21 in a p53-independent manner and reduces cell proliferation (82). Genetic alterations of this gene, such as deletions and loss of function have been found in a minority of high grade prostate cancers (82, 83).

i. CDKN2A (p16): Loss of the p16 gene was observed in a clinical specimen of metastatic prostate cancer (84).

j. ATFB1: It is located on 16q22 coding for a cell cycle active protein and has been recently implicated in prostate cancer. A study found that 36% of the tumors tested had missense mutations likely to inactivate the gene’s function (85). ATFB1 is a transcription factor regulating the expression of α-fetoprotein (86).

k. Annexins: They have been found by cDNA microarrays to be significantly downregulated in prostate cancer cell lines (87, 88). Their function is to maintain calcium homeostasis and regulating the cytoskeleton and cell motility.

2) Oncogenes: They derive from proto-oncogenes because of genetic damage. Proto-oncogenes participate in the normal growth and proliferation of cells. In case of genetic alteration (mutation, deletion etc.) they gain a new function that leads to abnormal cell growth and oncogenic properties. The following oncogenes have been found to play a role in prostate cancer:

a. c-MYC: The c-MYC proto-oncogene is a member of the basic helix-loop-helix-leucine zipper (bHLHZ) family of transcription factors. It encodes the c-MYC protein, which promotes cell proliferation and transformation (5, 89). Its amplification and overexpression was observed in 8% of primary prostate cancers and in about 30% of metastatic lesions (90, 91). Such overexpression is significantly correlated with Gleason grade and poor prognosis in advanced prostate cancer (91).

b. c-ErbB2 (Her-2 neu): It belongs to the epidermal growth factor receptor (EGFR) family. The c-ErbB2 gene encodes a transmembrane phosphoprotein. There is a controversy over the role of Her-2 neu in prostate cancer. There are studies that report overexpression of the gene in prostate cancer and some suggest that expression increases as prostate cancer progresses to androgen independence (92, 93). Other studies have not identified Her-2 neu amplification nor overexpression in prostate cancer (94, 95).

c. Bcl-2: This gene is not expressed in the normal prostate, but is commonly expressed in prostate and other primary cancers (96). It promotes cell survival through the inhibition of the apoptotic pathway (97). Bcl-2 has also been implicated in the development of androgen independent prostate cancer because of its increased expression in the advanced stages of disease (97, 98). It is suggested that androgen-mediated mechanisms may act through Bcl-2-mediated apoptotic pathways (99).The overexpression of Bcl-2 in prostate cancer safeguards the tumor cells from apoptosis (100, 101).

d. Prostate stem cell antigen (PSCA): It has been found to have an increased expression in 80% of prostate specimens in a recent study including high grade PIN (102). Higher PSCA expression is associated with increasing Gleason score, disease stage and progression to androgen independence (103). In an animal study the administration of anti-PSCA monoclonal antibodies decreased tumor growth and inhibited metastases providing promising results for future prostate cancer immunotherapy (104).

e. ERG and ETV1: These two genes belong to the ETS transcription factors and have been found to be overexpressed in prostate cancer tissues. They have been found both in primary and in metastatic disease. They seem to be activated by fusion to the TMPRSS2, which is a prostate-specific cell-surface serine protease gene, generating an androgen-responsive fusion oncoprotein (105, 106).

f. Hepsin: This protein is a membrane bound serum protease with an important role in cell growth. Expression of hepsin protein in prostate cancer has been found by cDNA microarrays to correlate inversely with patient prognosis (107).

g. PIM1: It encodes a protein kinase which was also found to be upregulated in prostate cancer by CDNA microarrays (107).

h. A-Methyl Coenzyme A racemase (AMACR): This gene product is involved in oxidation of fatty acids (108, 109) found in dairy products and beef, the consumption of which has been associated with increased risk of prostate cancer (110). Approximately 88% of prostate cancers in a study demonstrated higher AMACR staining (111). It may also be used by pathologists as prostate cancer tissue marker in biopsy specimens with 97% sensitivity and 100% specificity (109).

i. Androgen receptor (AR): In the prostate, AR is localized predominantly to nuclei of granular epithelial cells. The androgen receptor (AR) belongs to the superfamily of nuclear receptors. It is a member of the steroid-thyroid hormone receptor gene and it is coded by a gene located at X q11-q12 (111). Nuclear receptors are ligand-inducible transcription factors that mediate the signals of a broad variety of fat-soluble hormones, including the steroid and vitamin D3 hormones, thyroid hormones retinoids. AR can modulate gene expression directly by interacting with specific elements in the regulatory regions of target genes or indirectly by activating various growth factor signaling pathways. The nuclear receptor exerts its action by binding to cytoplasmic hormones and translocating to the nucleus where it interacts with basal transcription machinery to trigger the transcription of the target genes. AR mutations in un-treated prostate cancer are rare (112). Most metastatic androgen-independent prostate cancers express high levels of androgen-receptor gene transcripts. Taplin et al. demonstrated point mutations in the AR gene in metastatic cells (113). The resulting nucleotides derived from the corresponding changes in amino-acid sequence. Examples of point mutations such as CAA-to-CGA or GCC-to-ACC were sporadically detected in the AR gene in metastatic cells of prostate cancer. AR mutations are more common in patients that have been treated for prostate cancer with anti-androgens (112). Some studies have shown that the CAG repeat polymorphism length has an inverse relationship with transcriptional activity of the AR (5, 111). Short CAG length has been correlated with high grade, high stage metastatic prostate cancer. However, a recent epidemiological study by Zeegers et al demonstrated that the correlation of CAG allele length and prostate cancer is weak (114). Mutations, amplifications and deletions of the AR gene as well as conformational changes of the AR protein have been implicated in the development of androgen insensitivity. Such mutations of the AR gene have been identified in the LNCaP cell line, which has been associated with recurrence of prostate cancer (1). In addition to that, it has been shown that in LNCaP cell lines the interaction with estrogens stimulates their growth, in comparable levels to that of testosterone or DHT (115). In contrast, estrogens have a direct inhibitory effect on PC3 cell lines in prostate cancer, mediated by TGF1β. Other studies have shown that estradiol (E2) can directly bind to AR of DU145 cell lines of prostate cancer cells and enhance the transcriptional activity of AR.

j. CYP17: The CYP17 allele encodes for the cytochrome P-450c 17α enzyme that is responsible for the synthesis of testosterone. CYP17 allele mutations have been found in cases of sporadic and hereditary prostate cancers (5).

k. SRD5A2: Inside the prostatic cell testosterone is converted to 5-dihydrotestosterone (DHT). This action is mediated by the enzyme 5α-reductase, located mainly on the nuclear membrane of prostatic cells. The enzyme 5α-reductase is encoded by the allele SRD5A2, located on chromosome 2. DHT is the principal androgen in the growth of the normal and the hyperplastic prostate. Therefore polymorphisms of the SRD5A2 allele will affect functionality of DHT. Alleles that code for enzymes with increased activity have been shown to be associated with increased prostate cancer risk (116).

l. CYP3A4: The steroid hydroxylase CYP3A4 is the most abundant P-450 enzyme in the human liver and assists in the metabolism of a large number of prescription medications. It has been implicated in the development of breast and prostate carcinogenesis via the modulation of sex hormone metabolite levels. Two studies have shown that CYP3A4 polymorphisms to confer an increased risk of prostate cancer in men with benign prostatic hyperplasia. However, further studies have produced inconclusive results and the role of CYP3A4 remains yet to be elucidated (117).

m. Vitamin-D receptor (VDR): The vitamin D3 receptor (VDR) is an intracellular hormone receptor that specifically binds the active form of vitamin D (1, 25-dihydroxyvitamin D3 or calcitriol), which has been shown to promote differentiation and inhibit the proliferation of prostate cancer cells. It interacts with target-cell nuclei to produce a variety of biologic effects. It has been reported that there is an association between the most commonly recognized polymorphisms in the VDR variants such as Cdx2, Fokl, Bsml, Apal, Taql, and the poly-A microsatellite and prostate cancer (116). Recent studies have shown that only Fokl and Apal were associated with disease and Apal had only a very weak association (118).

n. Growth factors: Receptors for steroid and thyroid hormones are located inside target cells, in the cytoplasm or nucleus, and therefore they all have a common function as ligand-dependent transcription factors. The hormone-receptor complex then binds to promoter regions of responsive genes and stimulates or sometimes inhibits transcription from those genes. The net result is the production of polypeptides, amongst which are growth factors. The main growth factors are: IL-6 (interleukin-6), EGF (epidermal growth factor), IGF I&II (insulin-like growth factor), vascular endothelial and TGF-α,β (transforming growth factor). The epidermal growth factor receptor (EGFR) plays an important role in the development and progression of prostate cancer and its overexpression is associated with decreased survival. With progression, prostate cancer cells switch from epidermal growth factor (EGF) to transforming growth factor α (TGF-α) synthesis, which contributes to autocrine growth and unrestrained proliferation (119). Chan J.M. reported a strong positive association observed between circulating IGF-I levels and prostate cancer risk (120). Interleukin-6 (IL-6) is a cytokine that regulates growth, differentiation and apoptosis of various types of malignant tumors, including prostate carcinomas by activating the STAT and/or mitogen activated protein kinase (MAPK) signaling pathways. The levels of IL-6 are elevated in sera of patients with metastatic prostate cancer. Prostate cancer growth is accelerated in long-term exposure to IL-6 (121). Transforming growth factor β (TGF-β) is a multifunctional peptide that controls proliferation, differentiation, and other functions in many cell types, including prostate cancer cells. TGF-β acts synergistically with TGF-α in inducing cellular transformation. TGF-β and vascular endothelial growth factor stimulate also angiogenesis in the tumor site (122). Transforming growth factor (TGF)-β1 is a potent growth inhibitor of prostate epithelial cells, and aberrant function of its receptor type I and II correlates with tumor aggressiveness. However, intracellular and serum TGF-β1 levels are elevated in prostate cancer patients and further increased in patients with metastatic carcinoma (121).

Two other oncogenes have been recently recognized and may play a role as novel targets for molecular genetic intervention, or allow accurate prediction of the likelihood of progression of the tumor:

o. c-Kit/tyrosine kinase receptor: It is a strong activator of the Src family tyrosine kinases. A recent study revealed that human tr-KitmRNA and its encoded protein are expressed in prostatic cancer cells. The study also shows a correlation between tr-Kit expression and activation of the Src pathway in the advanced stages of the disease (123).

p. STAT5: This gene has been identified as a crucial survival factor for prostate cancer cells as well as a signal transducer and activator of transcription. Its activation is also associated with high histological grade of prostate cancer (124, 125).

## 5. Conclusions

The tools offered by molecular biology in prostate cancer may be useful in disease detection and prediction of biological behavior of such a common disease. Such tools may lead to early diagnosis and patient selection for treatment of a disease with such a diverse behavior. Although several genes and epigenetic events have been discovered to play a role in prostate cancer initiation and progression, further studies are required in order not only to diagnose and select patients with aggressive tumor for treatment but for creating agents that could prevent or treat prostate cancer at its molecular roots.
